# Impact of Severe COVID‐19 on Interstitial Lung Disease in Autoimmune Systemic Diseases

**DOI:** 10.1002/rcr2.70472

**Published:** 2026-09-22

**Authors:** Clodoveo Ferri, Marco de Pinto, Giuseppe Murdaca, Giacomo De Luca, Davide Mohammad Reza Beigi, Virginia Caira, Roberta Foti, Andrea Cito, Caterina Naclerio, Jessica Maria Elisa Luppino, Amelia Spinella, Martina Orlandi, Veronica Batani, Federica Lumetti, Francesco Prampolini, Giuseppe Varcasia, Rosario Foti, Valeria Riccieri, Florenzo Iannone, Alessandro Antonelli, Dilia Giuggioli, the COVID‐19 and Autoimmune Systemic Diseases Italian Study Group, Clodoveo Ferri, Vincenzo Raimondo, Dilia Giuggioli, Laura Gragnani, Serena Lorini, Lorenzo Dagna, Silvia Laura Bosello, Rosario Foti, Valeria Riccieri, Serena Guiducci, Giovanna Cuomo, Antonio Tavoni, Rossella De Angelis, Fabio Cacciapaglia, Elisabetta Zanatta, Franco Cozzi, Giuseppe Murdaca, Ilaria Cavazzana, Nicoletta Romeo, Veronica Codullo, Roberta Pellegrini, Giuseppe Varcasia, Maria De Santis, Carlo Selmi, Giuseppina Abignano, Maurizio Caminiti, Massimo L’Andolina, Domenico Olivo, Ennio Lubrano, Amelia Spinella, Federica Lumetti, Marco de Pinto, Giacomo De Luca, Veronica Batani, Piero Ruscitti, Teresa Urraro, Marcella Visentini, Silvia Bellando‐Randone, Elisa Visalli, Davide Testa, Gabriella Sciascia, Francesco Masini, Greta Pellegrino, Francesca Saccon, Eugenia Balestri, Giusy Elia, Silvia Martina Ferrari, Antonio Tonutti, Francesca Dall’Ara, Giuseppa Pagano Mariano, Giorgio Pettiti, Giovanni Zanframundo, Raffaele Brittelli, Vincenzo Aiello, Ylenia Dal Bosco, Roberta Foti, Ilenia Di Cola, Daniela Scorpiniti, Enrico Fusaro, Tommaso Ferrari, Pietro Gigliotti, Corrado Campochiaro, Francesca Francioso, Carlo Iandoli, Virginia Caira, Anna Linda Zignego, Salvatore D’Angelo, Franco Franceschini, Marco Matucci‐Cerinic, Roberto Giacomelli, Andrea Doria, Poupak Fallahi, Florenzo Iannone, Alessandro Antonelli

**Affiliations:** ^1^ Rheumatology Unit University Hospital of Modena and Reggio Emilia Modena Italy; ^2^ Rheumatology Unit, Casa di Cura Madonna dello Scoglio Cotronei KR Italy; ^3^ Department of Internal Medicine University of Genova Genova Italy; ^4^ Allergology and Clinical Immunology Unit, San Bartolomeo Hospital Sarzana Italy; ^5^ Unit of Immunology, Rheumatology, Allergy and Rare Diseases San Raffaele Scientific Institute Milan Italy; ^6^ Rheumatology Unit, Dipartimento di Scienze Cliniche Internistiche, Anestesiologiche e Cardiovascolari Sapienza University of Rome Rome Italy; ^7^ Rheumatology Unit, Castrovillari Hospital Castrovillari Italy; ^8^ Division of Rheumatology A.O.U. “Policlinico‐San Marco” Catania Italy; ^9^ Rheumatology Unit, Department of Precision and Regenerative Medicine and Ionian Area (DiMePre‐J) University of Bari Aldo Moro Bari Italy; ^10^ Departmental Unit of Rheumatology “Mauro Scarlato” Hospital Scafati Italy; ^11^ Department of Medical and Surgical Sciences for Children and Adults University of Modena and Reggio Emilia Modena Italy

**Keywords:** autoimmune systemic diseases, COVID‐19, interstitial lung disease, SARS‐CoV‐2 systemic sclerosis

## Abstract

The objective of this study was to assess the impact of severe COVID‐19 pneumonia on the course of interstitial lung disease (ILD) in autoimmune systemic diseases (ASD). We conducted a multicentre case series of 27 ASD patients (20 systemic sclerosis, 4 rheumatoid arthritis, 1 interstitial pneumonia with autoimmune features, 1 dermatomyositis, 1 eosinophilic granulomatosis with polyangiitis) hospitalised for severe COVID‐19. Clinical data, pulmonary function tests and HRCT before and after infection were analysed. Four patients died during acute COVID‐19. Among the 23 survivors, 10 developed new symptomatic ILD and 13 showed worsening of pre‐existing ILD. Six exhibited significant HRCT progression, including NSIP‐to‐UIP transition; one developed lung adenocarcinoma. Despite treatment, five more patients died during follow‐up from progressive ILD. Overall mortality approached one‐third, with systemic sclerosis most affected. Severe COVID‐19 may trigger or accelerate ASD‐related ILD. The observed NSIP‐to‐UIP shift highlights a multistep process potentially driven by SARS‐CoV‐2, underscoring the need for close ILD monitoring in ASD patients.

## Graphical abstract

In this multicentre case series of 27 patients with autoimmune systemic diseases, severe COVID‐19 pneumonia led to marked interstitial lung disease (ILD) worsening, with new‐onset or progressive ILD in all survivors and NSIP‐to‐UIP transition in six cases. Overall mortality reached 33%, and pre‐existing ILD emerged as the strongest adverse prognostic factor. These findings indicate that SARS‐CoV‐2 infection may act as a potent accelerator of fibrosing lung injury in autoimmune diseases—particularly systemic sclerosis—underscoring the need for vigilant monitoring and timely therapeutic intervention.

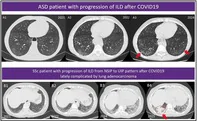

## 1 Introduction

Severe acute respiratory syndrome coronavirus 2 (SARS‐CoV‐2) infection causes coronavirus disease 2019 (COVID‐19), characterised by variable combinations of pneumonia and thromboembolic complications [1]. The impact of COVID‐19 in patients with autoimmune systemic diseases (ASD) has been widely investigated [2, 3]. These disorders—including chronic arthritis, connective tissue diseases and systemic vasculitides—are associated with increased susceptibility to infections due to immune dysregulation [1]. Pre‐existing interstitial lung disease (ILD) and/or diffuse vasculopathy confer a higher risk of severe COVID‐19 and poorer outcomes [4, 5, 6]. Among ASD, systemic sclerosis (SSc) is one of the most severe conditions, frequently complicated by ILD and widespread vasculopathy resulting from immune‐mediated fibroblast and endothelial injury. Rheumatoid arthritis (RA), although less frequently associated with ILD, may likewise predispose to more severe pulmonary complications during COVID‐19. At the onset of the pandemic in early 2020, the Italian multicentre COVID‐19 and ASD Study Group initiated a telemedicine‐based monitoring system to closely follow ASD patients [7]. Several observational studies later demonstrated the detrimental prognostic role of concomitant ILD and the reduced immunogenicity of COVID‐19 vaccines in patients receiving immunomodulatory therapies [4, 5, 8]. Here, we report a multicentre case series of ASD patients who developed severe COVID‐19 pneumonia requiring hospitalisation over a five‐year period, focusing on the impact of SARS‐CoV‐2 infection on the onset or progression of ILD during long‐term follow‐up.

## 2 Case Series

Twenty‐seven ASD patients (22 females, 5 males; mean age 58.5 ± 12.3 years; mean disease duration 9.9 ± 4.8 years) were consecutively evaluated at seven tertiary referral centres between March 2020 and February 2025 (Table 1). Diagnoses included: SSc (20), seropositive RA [4], interstitial pneumonia with autoimmune features [1], dermatomyositis [1] and eosinophilic granulomatosis with polyangiitis [1]. ILD assessment was based on dyspnea, cough, pulmonary function tests, 6‐min walking test (6MWT) and high‐resolution CT (HRCT). Radiologic patterns—nonspecific interstitial pneumonia (NSIP) and usual interstitial pneumonia (UIP)—were classified according to standard criteria. SARS‐CoV‐2 infection was confirmed by PCR on nasopharyngeal swab.

**TABLE 1:** COVID‐19 and lung involvement in 27 patients with autoimmune systemic diseases (ASD).

Pt no. diagnosis, age‐sex	Lung involvement^a^			Outcome/comments
	Before COVID‐19^a^	After COVID‐19 (year)	End of FU^b^	
1. lcSSc, 80F	Mild NSIP, mild dyspnea	2023 (April): worsening NSIP, PAH	NSIP & PAH	*Deceased* Severe ILD & PAH complicated by pneumococcal pneumonia (Dec. 2023)
2. dcSSc, 45M	Mild NSIP, mild dyspnea	2020: severe COVID‐19, ARSD	—	*Deceased* Severe COVID‐19 pneumonia with ARSD unresponsive to combined therapies: CS, anti‐IL6, antivirals; vaccine not available (2020)
3. lcSSc, 66F	Mild NSIP, mild dyspnea	2021: severe COVID‐19, ARSD no‐vax patient	—	*Deceased* Severe COVID‐19 pneumonia with ARSD unresponsive to combined therapies: CS, anti‐IL6, antivirals
4. dcSSc, 63F	Mild NSIP, mild dyspnea	2022, worsening NSIP, dyspnea at rest; no‐vax patient	Dec 2024, progression from NSIP to UIP	Severe ILD unresponsive to treatments (MMF, nintedanib) with episodes of asthma treated with dupilumab
5. IPAF, 78M	Mild NSIP and dyspnea	2023, severe pneumonia, UIP	Stable UIP	Progression HRCT alterations from NSIP to UIP after COVID‐19. Stabilisation of symptoms and radiological UIP alterations with nintedanib and O2 therapy
6. dcSSc, 51F	Mild NSIP since 2013, asymptomatic	2022, dyspnea at exertion, cough, UIP	UIP	Stable respiratory symptoms after 6‐month therapy with nintedanib; January 2025: detection of lung adenocarcinoma at HRCT
7. RA, 67M	Very mild asymptomatic NSIP	2021: Mild dyspnea at exertion, FVC from 100 to 80%, UIP	UIP with dyspnea at rest, FVC 65%	*Deceased* in Jul 2024 due to a second episode of severe COVID‐19, after Initial stabilisation with 11‐month nintedanib therapy
8. dcSSc, 60F	Mild NSIP asymptomatic	2023: dyspnea at exertion, fibrotic NSIP	Stable	Appearance of fibrotic ILD with bronchiectasis after COVID‐19, stable with long‐term MTX therapy
9. lcSSc/SS, 60F	NSIP, mild exertional dyspnea	2024: dyspnea at rest, UIP	Stable UIP	Chronic dyspnea, second COVID‐19 episode within 2024; February 2025: detection of thymoma at HRCT
10. dcSSc, 70F	No ILD	2024 (Oct): mild dyspnea at exertion, mild NSIP	Stable NSIP	Mild NSIP with exertional dyspnea, stabilisation with MMF therapy (6 min.w.test from 180 to 320 m)
11. dcSSc, 53F	Mild NSIP, mild exertional dyspnea	2024 (Oct): dyspnea at rest, dry cough	Stable NSIP	Improved dyspnea with MMF therapy, 6 min.w.test from 200 to 400 m
12. dcSSc, 44F	No ILD	2024 (March): Dyspnea at rest, dry cough	Mild NSIP	*Deceased* Oct 2024 due to acute pulmonary edema secondary to heart failure, after initial stabilisation of ILD with MMF & rituximab
13. dcSSc, 58F	No ILD	2024 (Feb). Dyspnea at rest, dry cough	Worsening NSIP	Progressive worsening of ILD: severe dyspnea at rest needing home oxygen therapy, unresponsive to ongoing treatments (MMF & rituximab)
14. dcSSc, 64F	No ILD	2024 (Apr): Dyspnea at rest, dry cough	Worsening NSIP	*Deceased* Nov 2024 due to progressive deterioration of lung function unresponsive to MMF and rituximab treatments
15. dcSSc, 67F	No ILD	2024 (Jan): Dyspnea at rest, dry cough	Stable NSIP	Stable ILD with exertional dyspnea, improved 6 min.w.test (from 190 to 310 m) with MMF and rituximab treatments
16. RA, 45F	No ILD	2024 (Sep): Dyspnea at rest, dry cough	Stable NSIP	Stabilisation of ILD with tocilizumab & MMF, improved 6 min.w.test from 180 to 380 m
17. RA, 49F	No ILD	2024 (Jun): Dyspnea at rest, dry cough	Stable NSIP	Stabilisation of ILD with MMF therapy with improving respiratory function symptoms, 6 min.w.test from 220 to 360 m
18. RA, 43F	No ILD	2024 (Sep): Dyspnea at rest, dry cough	Stable NSIP	Stabilisation of ILD with MMF therapy respiratory symptoms improved 6 min.w.test from 200 to 420 m
19. DM, 50M	No ILD	2024 (Jul): Dyspnea at rest, dry cough	Stable NSIP	Stabilisation of ILD with MMF therapy respiratory symptoms improved 6 min.w.test from 150 to 380 stabilisation
20. dcSSc, 34F	Mild NSIP, mild exertional dyspnea	2021: worsening of symptomatic ILD	Worsening of ILD, from NSIP to UIP	Marked progression of ILD, patient unresponsive to treatment with MMF+ nintedanib (2022) + anti‐IL6 (2024)
21. lcSSc, 48F	No ILD	2023: exertional dyspnea	Fibrosing NSIP	Stabilisation of ILD, patient undergoing RTX cycles
22. dcSSc, 64F	Cough, very mild NSIP	2022: worsening dyspnea and HRCT findings (NSIP)	Stable fibrosing NSIP	Stabilisation of ILD with ongoing nintedanib treatment during the last year (2024)
23. EGPA, 42M	Mild dyspnea, cough NSIP	2022: marked worsening of NSIP findings; no‐vax patient	—	*Deceased* Dec 2022 due to marked progression of ILD at HRCT after severe COVID‐19
24. dcSSc, 65F	Mild dyspnea at exertion, NSIP	2022 (March): worsening dyspnea, dry cough	Stable NSIP	Stabilisation of ILD with MMF & rituximab treatments (HRCT Nov 2924)
25. dcSSC, 74F	Mild dyspnea at exertion, NSIP	2023 (March): worsening dry cough, dyspnea, HRCT lesions	Stable NSIP	Stabilisation of ILD with MMF treatment
26. lcSSc, 75F	Dyspnea, dry cough NSIP	2022: severe COVID‐19 with ARSD	—	*Deceased* Patient deceased (2022) due severe ARSD complicating COVID‐19 pneumonia
27. lcSSc, 65F	Dyspnea, mild NSIP GERD, UD	2021: severe COVID‐19, worsening dyspnea	Worsened fibrosing NSIP	*Deceased* due to ascitic decompensation complete portal thrombosis (Dec 2023); fibrosing NSIP unresponsive to nintedanib

Abbreviations: ARSD, acute respiratory distress syndrome; dcSSc, diffuse cutaneous SSc; lcSSc, limited cutaneous SSc; NSIP, non‐specific interstitial pneumonia; PAH, pulmonary arterial hypertension; UIP, usual interstitial pneumonia.

^a^ Evaluated on the basis of symptoms (dyspnea and/or cough), spirometry, 6 min/w.test and high‐resolution computed tomography (HRCT).

^b^ Last control for surviving patients (Dec 2024–Feb 2025).

### 2.1 ILD Before COVID‐19

Within the 12 months preceding COVID‐19, ILD was documented in 17/27 patients, all presenting NSIP; 14 had mild‐to‐moderate respiratory symptoms. Ten patients had no ILD before COVID‐19. Regarding vaccination status, 23 patients were vaccinated; three refused vaccination (patients 3, 4, 23), and one died before vaccine availability (patient 2).

### 2.2 Early Effects of COVID‐19 on Lung Involvement

All 27 patients developed at least one episode of COVID‐19 requiring hospitalisation and aggressive treatment (high‐dose corticosteroids, IL‐6 inhibitors, oxygen therapy and/or antivirals). Four patients (2, 3, 23, 26) died during the initial episode due to severe pneumonia. Among the 23 survivors, all showed ILD progression on post‐COVID HRCT, including the 10 previously ILD‐free patients. A transition from NSIP to UIP occurred in 4 patients shortly after COVID‐19 (patients 5, 6, 7, 9).

### 2.3 Long‐Term Follow‐Up

**FIGURE 1:**
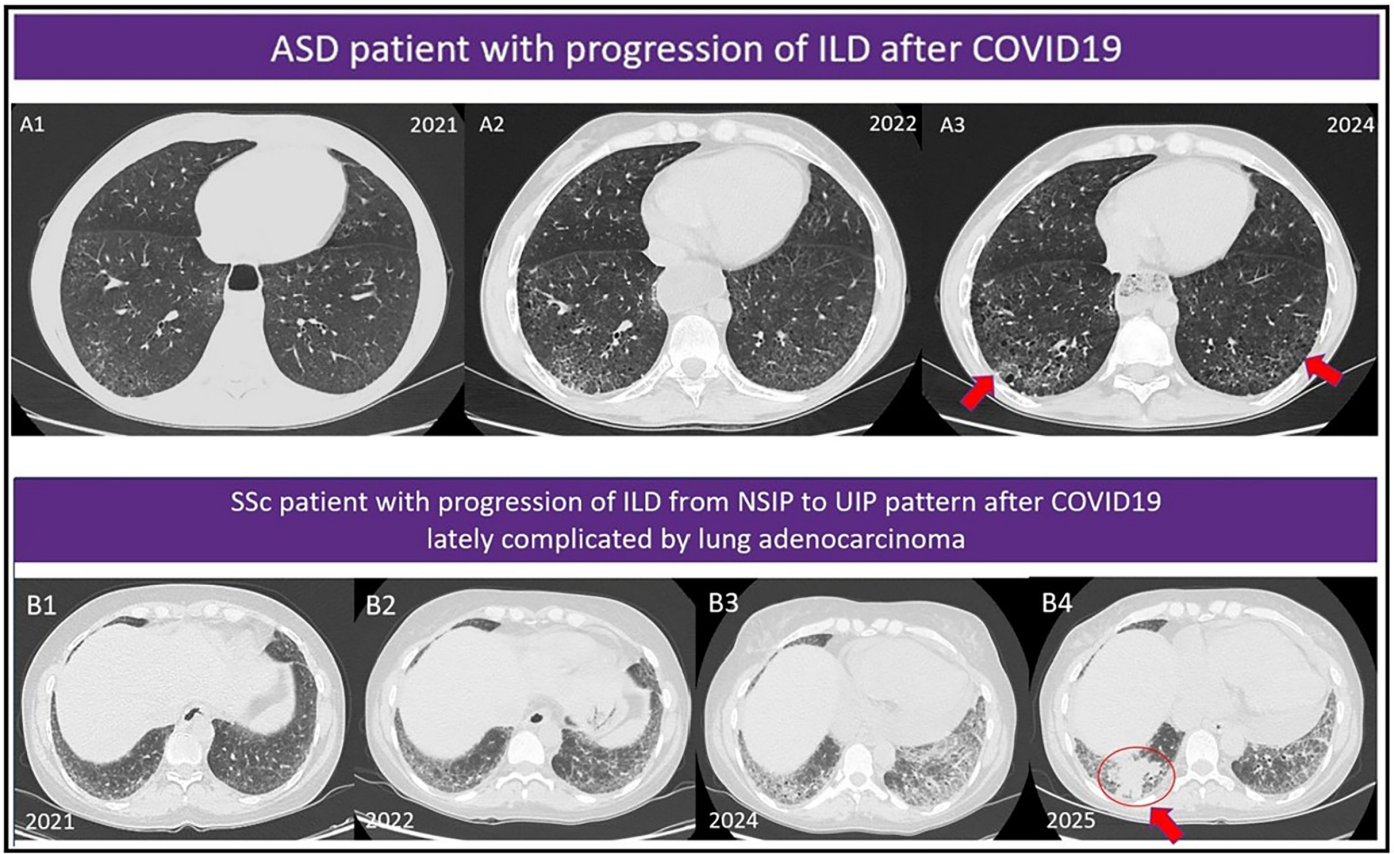
(A) SSc patient with progression of ILD after COVID‐19 (patient no. 20, Table 1). Before COVID‐19 (A1) mild dyspnea at exertion: at HRCT subtle GGO and fine reticular pattern in subpleural areas of the inferior lobes that involve < 20% of the lungs (fibrotic NSIP). Initial treatment with mycophenolate mofetil. The clinical, functional and radiological condition remained stable until 2021. After COVID‐19 (A2) worsening of cutaneous, vascular and pulmonary status, with increase, at HRCT, of interstitial thickening and more evident reticular opacities that involve > 20% of the lungs, in addition to appearance of traction bronchiolectasis. In May 2022 treatment with nintedanib was started. At the end of the follow‐up (2024, A3) dyspnea at rest with onset of cardiac involvement, tocilizumab was added to the regimen in February 2024. At HRCT, widespread areas of reticular thickening of the interstitium on a fibrotic basis, traction bronchiolectasis and distortion of the interstitial web with cystic changes with initial honeycombing pattern (red arrows). These findings, dominant in the subpleural and basal regions, are compatible with UIP pattern. (B) SSc patient with progression of ILD from NSIP to UIP pattern after COVID‐19 lately complicated by lung adenocarcinoma (patient no. 6, Table 1). Before COVID‐19 (B1), patient clinically asymptomatic with stable HRCT findings since 2013, namely subtle ground‐glass opacities (GGO) and fine reticular opacities in subpleural areas with basal predominance (mild ILD—NSIP pattern). After the first episode of COVID‐19 (B2), dyspnea at exertion, cough and HRCT scan showing an increase of GGO and interstitial reticular thickening in addition to development of subtle traction bronchiolectasis. After the second episode of COVID‐19 (B3), dyspnea at rest, cough and recurrent fever with further increase of interstitial thickening and extension of GGO at HRCT (prevalence of basal subpleural region, suggestive of UIP pattern): the clinical picture stabilises after a 6‐month therapy with nintedanib. In January 2025 (B4), worsening of clinical conditions and appearance of a nodular consolidation in the right lower lobe, which was found to be lung adenocarcinoma on transthoracic biopsy (red arrow).

During extended follow‐up, an additional two patients (4, 20) progressed to UIP, yielding a total of 6/23 (26%) transitions from NSIP to UIP (Figure 1). After the acute episode, 21/23 survivors received treatment aimed at limiting further ILD progression—mycophenolate mofetil (MMF), corticosteroids, IL‐6 inhibitors, rituximab and/or nintedanib. ILD stabilisation or improvement was observed in 16 patients, whereas 5 remained unresponsive (patients 4, 13, 14, 20, 27). Two individuals developed severe oncologic complications at the end of follow‐up: lung adenocarcinoma (patient 6) and thymoma (patient 9). Patient 12 died from acute pulmonary edema secondary to heart failure; patient 27 died from complications of portal thrombosis. A second COVID‐19 episode occurred in two patients (7, 9), with one death (patient 7). Among unvaccinated individuals, three died (2, 3, 23) and one (patient 4) developed severe ILD progression unresponsive to MMF and nintedanib.

### 2.4 Overall Outcomes

One‐third of the entire cohort died (9/27, 33%): 7/20 SSc, 1/4 RA and 1 EGPA. Four deaths occurred during the first COVID‐19 episode and five during follow‐up due to ILD progression and/or cardiopulmonary complications. No significant demographic differences were found between survivors and non‐survivors. Pre‐existing ILD emerged as a major prognostic factor: 12/17 (70.6%) patients with prior ILD experienced death and/or progression to UIP versus 2/10 (20%) without ILD (*p* = 0.0183; OR = 8.70; 95% CI 1.17–113.1).

## 3 Discussion

This multicentre case series highlights the severe long‐term impact of COVID‐19 pneumonia in patients with ASD, with high rates of ILD worsening and mortality, especially among those with pre‐existing lung involvement. COVID‐19 led to a change in radiological pattern from NSIP to UIP in six patients, occurring either soon after the acute infection or during prolonged follow‐up. Overall mortality reached 33%, mostly attributable—directly or indirectly—to COVID‐19–related pulmonary injury. Although many patients achieved ILD stabilisation with immunomodulatory and antifibrotic therapies, the development of late complications—including lung cancer and thymoma—underscores the vulnerability of this population. Despite widespread vaccination, adverse events remained frequent, although unvaccinated patients experienced particularly severe outcomes (three deaths and one severe ILD progression).

ILD is one of the most harmful determinants of prognosis in ASD. Its prevalence varies across disorders but is particularly high in SSc. Environmental insults—including viral infections—may accelerate ILD progression and SARS‐CoV‐2 appears to be a potent trigger [9]. COVID‐19 and SSc share core pathogenetic mechanisms: inflammatory interstitial pneumonia evolving into fibrosis and microvascular injury leading to widespread endothelial dysfunction [9]. These similarities may explain the marked ILD deterioration observed in SSc patients after severe COVID‐19.

Pre‐existing ILD has consistently been identified as a strong adverse prognostic factor in COVID‐19 across multiple studies [1, 4, 5, 10, 11, 12]. Nonetheless, some reports indicated comparable mortality between ASD patients and the general population during earlier pandemic phases [7], perhaps due to the protective effect of chronic immunosuppression in mitigating the cytokine‐storm–mediated lung injury.

The development of pulmonary arterial hypertension or lung cancer in some SSc patients aligns with known long‐term complications of the disease [9].

Evidence regarding long‐term ILD evolution after COVID‐19 remains limited and variable. Some studies suggested that COVID‐19 does not invariably worsen ILD in vaccinated SSc patients [13], whereas others documented significant long‐term deterioration in nearly half of ILD cases [6]. Population‐based analyses confirmed that ILD markedly increases COVID‐19 mortality irrespective of ASD status [4]. Additional reports emphasised the fragility of ILD associated with RA [11], inflammatory myopathies [14] and SSc [10].

Post‐COVID pulmonary fibrosis in the general population is increasingly recognised: up to 30% of hospitalised patients develop persistent interstitial lung abnormalities, and about one‐third exhibit irreversible fibrosis [15].

The patients described here likely represent the severe extreme (‘tip of the iceberg’) of a broader phenomenon observed in ASD populations [13].

Key insights from this case series include: (I) severe COVID‐19 pneumonia may accelerate ILD progression and trigger transformation from NSIP to UIP; (II) SSc patients appear particularly susceptible due to pathogenetic overlap with COVID‐19 and high baseline ILD prevalence.

Overall, these findings reinforce the multifactorial, multistep nature of ILD progression and highlight the need for vigilant monitoring and aggressive management of ASD patients experiencing severe COVID‐19.

## Author Contributions

Clodoveo Ferri: conceived the study, contributed to data collection and analysis and participated in manuscript writing and creation of figures and tables. Marco de Pinto: participated in patient management, provided clinical data, contributed to manuscript writing and created figures and tables. Giuseppe Murdaca, Giacomo De Luca, Davide Mohammad Reza Beigi, Virginia Caira, Roberta Foti, Andrea Cito, Caterina Naclerio, Jessica Maria Elisa Luppino, Amelia Spinella, Martina Orlandi, Veronica Batani, Federica Lumetti, Giuseppe Varcasia, Rosario Foti, Valeria Riccieri: all participated in patient management, provided clinical data and contributed to the interpretation of findings and critical revision of the manuscript. Francesco Prampolini: performed expert HRCT assessment for included cases and contributed to the creation of imaging figures. Florenzo Iannone: promoter of the Targeted Research Project, contributed to patient management, data interpretation and critical manuscript revision. Alessandro Antonelli: promoter of the Targeted Research Project, contributed to data interpretation and critical manuscript revision. Dilia Giuggioli: promoter of the Targeted Research Project, contributed to study design, patient management, data interpretation and critical manuscript revision.

## Funding

This work was supported by the Targeted Research Project RF‐2021‐12374986 CUP Master D55E22000670001, funded by the Ministero della Salute—Targeted Research Call 2021, Ministerial Decree dated 12.11.2021. Participating Institutions: Azienda Ospedaliero‐Universitaria Pisana, Azienda Ospedaliero‐Universitaria Bariand Azienda Ospedaliero‐Universitaria Modena. Institutional Recipient: Tuscany Region.

## Consent

The authors declare that written informed consent was obtained from all the patients for the publication of this manuscript and accompanying images and attest that the form used to obtain consent from the patients complies with the Journal requirements as outlined in the author guidelines.

## Conflicts of Interest

The authors declare no conflicts of interest.

## Data Availability

The authors have nothing to report.

## References

[1] A. Strangfeld , M. Schäfer , M. A. Gianfrancesco , et al., “Factors Associated With COVID‐19–Related Death in People With Rheumatic Diseases: Results From the COVID‐19 Global Rheumatology Alliance Physician‐Reported Registry,” Annals of the Rheumatic Diseases 80, no. 7 (2021): 930–942. 10.1136/annrheumdis-2020-219498 PMC7843211 33504483

[2] C. Ferri , D. Giuggioli , V. Raimondo , P. Fallahi , and A. Antonelli , “COVID‐19 in Italian Patients With Rheumatic Autoimmune Systemic Diseases,” Annals of the Rheumatic Diseases 82, no. 9 (2023): e211. 10.1136/annrheumdis-2020-219113 33055077

[3] A. Antonelli , P. Fallahi , G. Elia , et al., “Effect of the COVID‐19 Pandemic on Patients With Systemic Rheumatic Diseases,” Lancet Rheumatology 3, no. 10 (2021): e675–e676. 10.1016/S2665-9913(21)00243-5 PMC8297993 34316725

[4] K. Miyashita , H. Hozumi , K. Furuhashi , et al., “Impact of Preexisting Interstitial Lung Disease on Mortality in COVID‐19 Patients From the Early Pandemic to the Delta Variant Epidemic: A Nationwide Population‐Based Study,” Respiratory Research 25, no. 1 (2024): 95. 10.1186/s12931-024-02723-3 PMC10880313 38383463

[5] C. Ferri , D. Giuggioli , V. Raimondo , et al., “COVID‐19 and Rheumatic Autoimmune Systemic Diseases: Role of Pre‐Existing Lung Involvement and Ongoing Treatments,” Current Pharmaceutical Design 27, no. 41 (2021): 4245–4252. 10.2174/1381612827666210903103935 34477509

[6] E. Martínez‐Besteiro , M. Molina‐Molina , A. M. Gaeta , et al., “Impact of COVID‐19 Infection on Patients With Preexisting Interstitial Lung Disease: A Spanish Multicentre Study,” Archivos de Bronconeumología 59, no. 4 (2023): 273–276. 10.1016/j.arbres.2023.01.001 PMC9817335 36732159

[7] C. Ferri , D. Giuggioli , V. Raimondo , et al., “COVID‐19 and Rheumatic Autoimmune Systemic Diseases: Report of a Large Italian Patient Series,” Clinical Rheumatology 39, no. 11 (2020): 3195–3204. 10.1007/s10067-020-05334-7 PMC7450255 32852623

[8] C. Ferri , V. Raimondo , D. Giuggioli , et al., “Impact of COVID‐19 and Vaccination Campaign on 1,755 Systemic Sclerosis Patients During First Three Years of Pandemic,” Journal of Translational Autoimmunity 7 (2023): 100212. 10.1016/j.jtauto.2023.100212 PMC10580042 37854035

[9] R. Swarnakar , Y. Garje , N. Markandeywar , and S. Mehta , “Exploring the Common Pathophysiological Links Between IPF, SSc‐ILD and Post‐COVID Fibrosis,” Lung India 39, no. 3 (2022): 279–285. 10.4103/lungindia.lungindia_89_22 PMC9200204 35488687

[10] C. Ferri , D. Giuggioli , V. Raimondo , et al., “COVID‐19 and Systemic Sclerosis: Clinicopathological Implications From Italian Nationwide Survey Study,” Lancet Rheumatology 3, no. 3 (2021): e166–e168. 10.1016/S2665-9913(21)00007-2 PMC7836813 33521657

[11] G. Figueroa‐Parra , E. L. Gilbert , M. O. Valenzuela‐Almada , et al., “Risk of Severe COVID‐19 Outcomes Associated With Rheumatoid Arthritis and Phenotypic Subgroups,” Lancet Rheumatology 4, no. 11 (2022): e765–e774. 10.1016/S2665-9913(22)00227-2 PMC9472567 36118532

[12] C. Ferri , V. Raimondo , L. Gragnani , et al., “Prevalence and Death Rate of COVID‐19 in Autoimmune Systemic Diseases in the First Three Pandemic Waves,” Current Pharmaceutical Design 28, no. 24 (2022): 2022–2028. 10.2174/1381612828666220614151732 35726427

[13] S. Panopoulos , V. Tzilas , V. K. Bournia , et al., “COVID‐19 and Protection of Vaccination in Patients With Systemic Sclerosis–Associated Interstitial Lung Disease,” Journal of Scleroderma and Related Disorders 8, no. 2 (2023): 113–119. 10.1177/23971983221143252 PMC9755035 37284697

[14] Y. Li , X. Tian , C. Sun , et al., “Outcome of COVID‐19 in Patients With Idiopathic Inflammatory Myopathy During the Omicron Wave in China,” PLoS One 20, no. 2 (2025): 64–69. 10.1371/journal.pone.0317319 PMC11809795 39928605

[15] M. Robertshaw and C. D. Kershaw , “Post‐COVID Interstitial Lung Abnormalities: Incidence and Management,” Current Pulmonology Reports 12, no. 2 (2023): 64–69. 10.1007/s13665-023-00307-y PMC10098239 37206298

